# Supplementary material for: Efficient coding of natural images in the mouse visual cortex
Source: Nat Commun. 2024 Mar 19;15:2466. doi: 10.1038/s41467-024-45919-3 (PMC10951403; doi:10.1038/s41467-024-45919-3)
Supplement: Supplementary file 1 — Supplementary Information [file 41467_2024_45919_MOESM1_ESM.pdf]

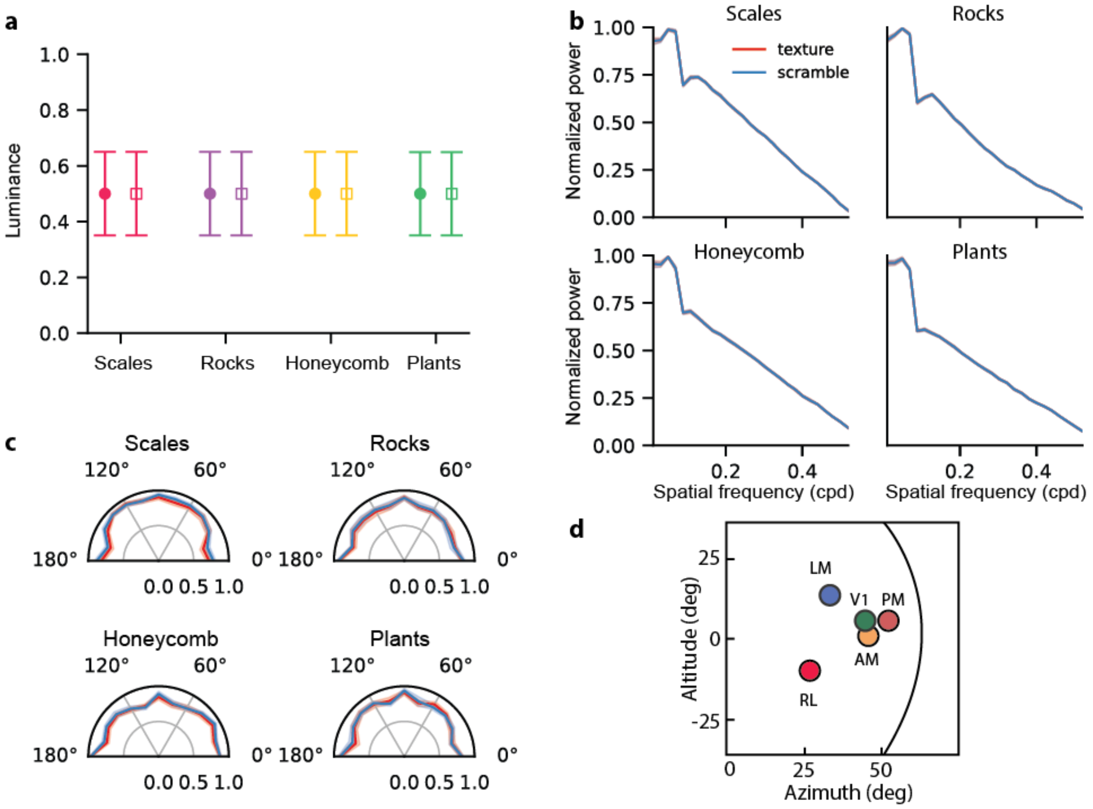

Supplementary Figure 1 | Characterization of the lower-order statistics of texture and scramble stimuli.

a, The mean luminance across pixels for all texture exemplars (filled circles) and scramble exemplars (open squares) for each family (color code as in the main figures). The error bars indicate the RMS contrast–that is, the standard deviation of the pixel intensities averaged across all exemplars; n = 20 images per family and stimulus type. **b**, The normalized spatial frequency power spectrum for each family—that is, the mean of the spectra computed for each exemplar, plotted in a frequency interval of maximum perceptual sensitivity for mice (0.02 - 0.5 cpd). **c**, The average orientation power for each texture family computed as the mean across exemplars for textures (red) and scrambles (blue). **d**, The mean azimuth and elevation of each reliably segmented visual area based on retinotopic mapping (filled dots, arbitrary colors; replotted from Garrett et al., J. Neurosci. 2014, Fig. 6E); the semicircle delineates the size (fixed across experiments) of the visual stimuli. Source data are provided as a Source Data file.

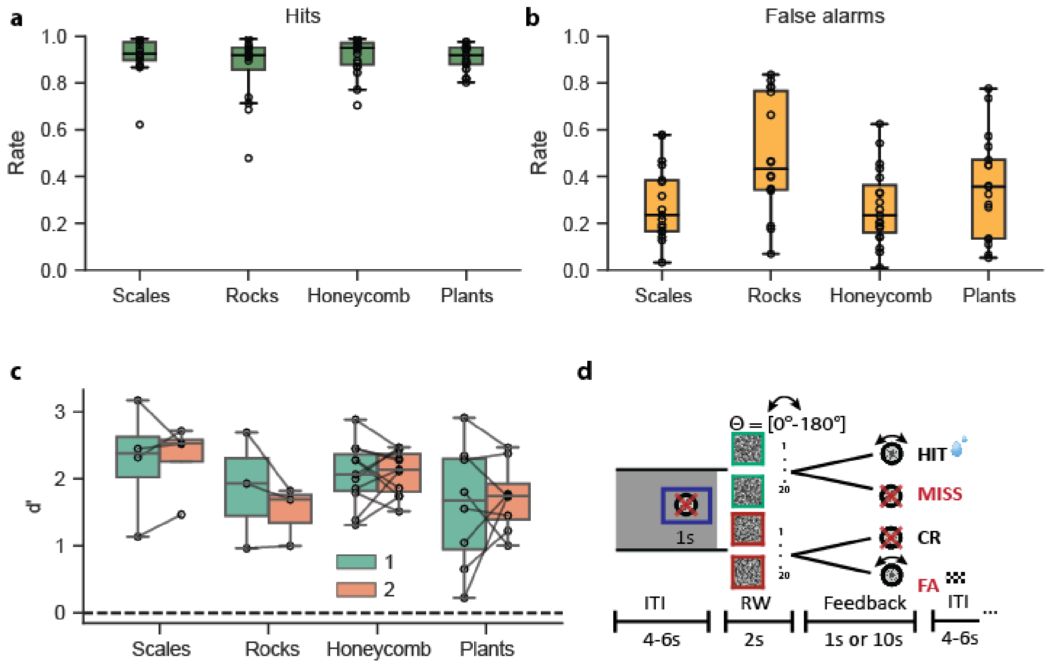

Supplementary Figure 2 | Performance of mice in the texture–scramble go/no go task.

a, The average hit rates for each texture family for the mice shown in Fig. 1f; each dot is for one animal. Box plots indicate the median with a horizontal bar; the box height denotes the inter-quartile range (IQR, 1st and 3rd quartile) and the whiskers extend by 1.5 x IQR. **b**, Same as in (a) but for false-alarm rates; boxplots as in (a). **c**, The performance (d') of mice (n = 3) for each texture–scramble pair over the last five sessions prior to a change in the set of 20 exemplars (green) and over the five sessions after the change (orange). Each dot indicates a training session; all differences are not significant; box plots as in (a). **d**, The texture-texture go/no-go task: in "go" trials mice need to rotate a rubber wheel with their front paws when shown a texture exemplar from a "go" family in order to obtain a water reward. In "no-go" trials, mice should refrain from making any wheel rotation when shown texture exemplar from a "no-go" family. ITI denotes the inter-trial interval; RW, the response window; and Feedback, the feedback period (water reward or high-contrast flickering grating). Each of the texture exemplars were randomly rotated between 0 and 180 degrees to prevent mice from solving the task using simple orientation cues. Source data are provided as a Source Data file.

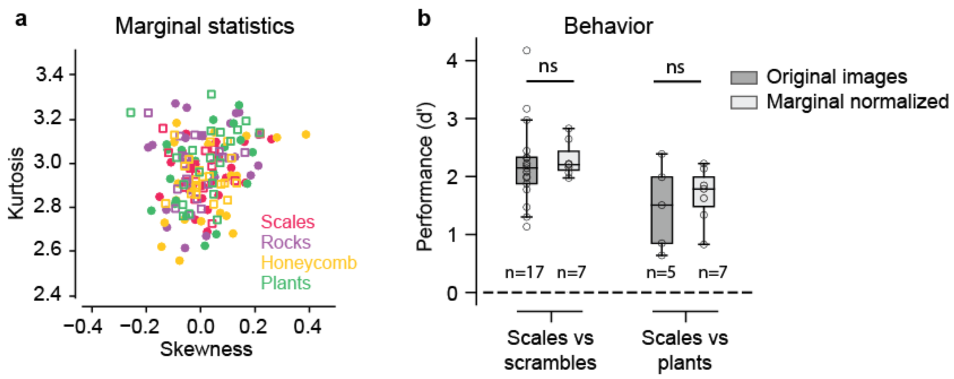

Supplementary Figure 3 | Behavioral discriminability with matching skewness and kurtosis.

a, For each of the four families, we generated a set of 20 exemplars in which we equalized pixel-amplitude histograms; images were then scaled and vignetted (Methods). Dots and squares show the resulting values for skewness and kurtosis (marginal statistics) for textures and scrambles. **b**, Behavioral discriminability (d') of expert mice in texture-scramble (scales) and texture-texture (scales – plants) tasks; dark gray boxes when using the original images, light gray boxes for images with matched marginal statistics. Each dot is for a mouse; significance was computed using a two-sided unpaired t-test. Box plots indicate the median with a horizontal bar; the box height denotes the inter-quartile range (IQR, 1st and 3rd quartile) and the whiskers extend by 1.5 x IQR. Source data are provided as a Source Data file.

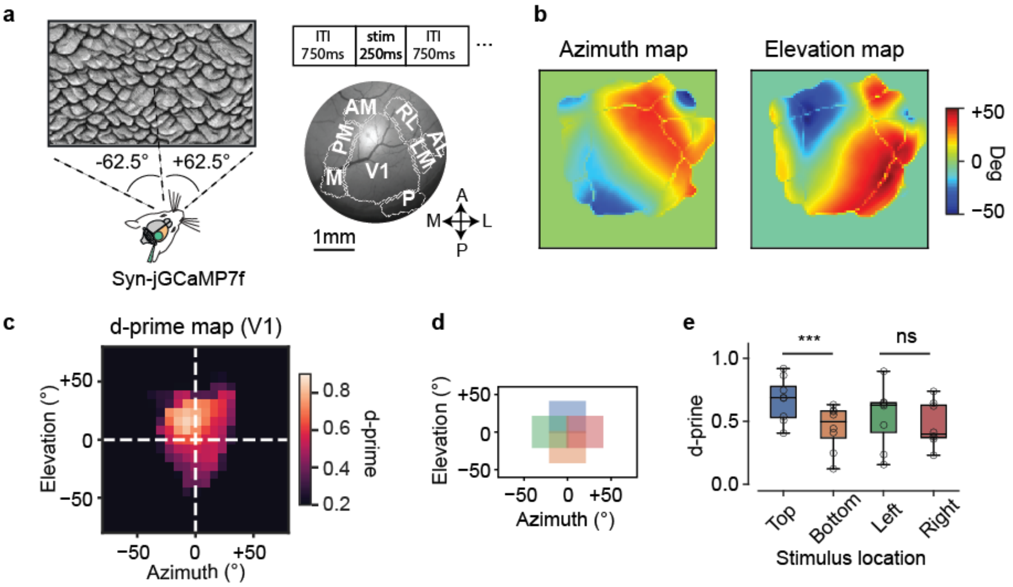

Supplementary Figure 4 | Texture discriminability gradient in V1.

a, Schematic figure of the mouse position during imaging experiments with full-field texture stimuli. The mouse was positioned so that its body midline pointed at the right edge of the monitor. The monitor had a coverage of 125º in azimuth and 97º in elevation. The visual stimuli were shown with the same timing parameters as in the other texture mapping experiments (250 ms stimulus on, 750 ms gray screen). Right: example of an unprocessed camera frame with segmented visual areas (labels as in Fig. 2a). **b**, Example azimuth and elevation sign-maps. **c**, Using the azimuth and elevation maps of each mouse, we measured the average d' values (textures vs scrambles) for each location in the visual space (azimuth, elevation) in area V1. The map shown was obtained from the average of n = 4 mice, 2 sessions per mouse. **d**, To test for the presence of a gradient in texture selectivity, we computed average d' values for each of the four illustrated quadrants (arbitrary colors). **e**, Average d' values for each of the 8 sessions for the upper, lower, right and left visual field. The upper visual field was significantly different from the lower visual field ($p = 5 \times 10^{-4}$, two-sided paired t-test, n = 8, 4 mice, 2 sessions per mouse). Each dot corresponds to the average d' for a particular mouse and session. Color code as in panel (d). Box plots indicate the median with a horizontal bar; the box height denotes the inter-quartile range (IQR, 1st and 3rd quartile) and the whiskers extend by 1.5 x IQR. Source data are provided as a Source Data file.

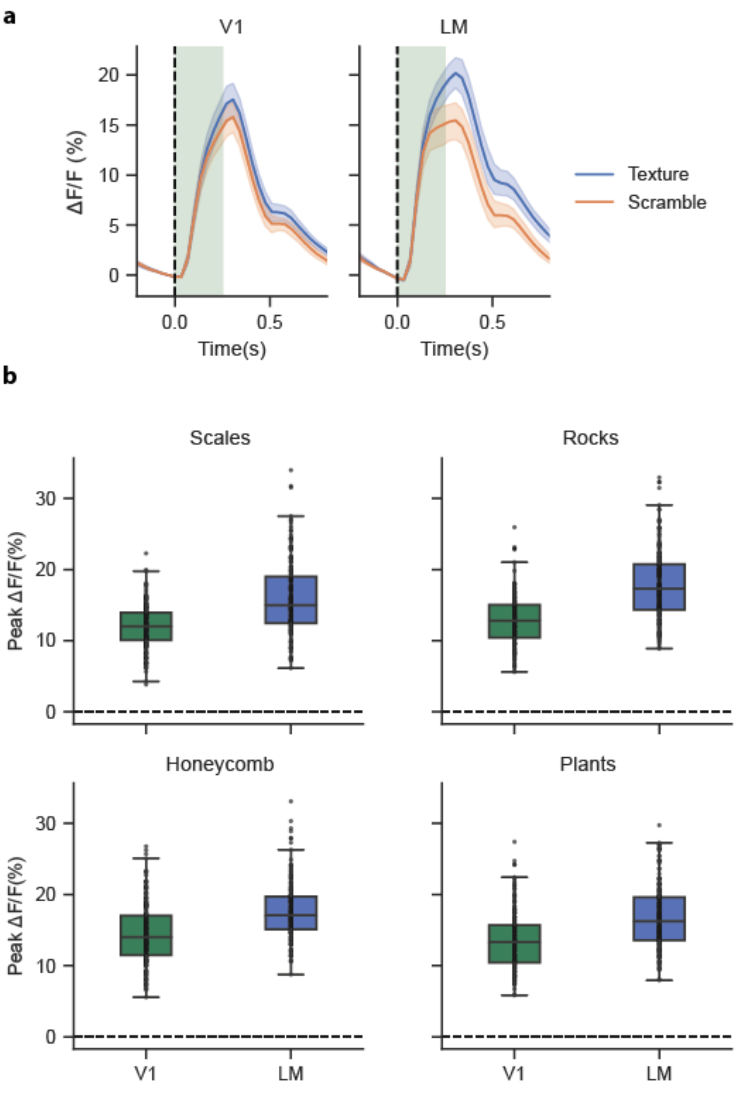

Supplementary Figure 5 | Population responses to visual stimuli derived from single-cell activity.

a, The mean population activity of all the visually responsive cells (solid line, average across all mice; error bands, 95% confidence intervals across the average response per mouse, n = 10) as a measure of the collective population response to textures and scrambles for the V1 and LM experiments. The dotted vertical line indicates the stimulus onset, whereas the green band indicates the stimulus duration. The mean population activity was computed as the mean response to all the textures and scrambles, averaging across repeats, rotations, exemplars, and all the responsive cells. **b**, Population responses were calculated the same way as in (a), but the responses were separated across the families (4 plots), and only the texture stimuli responses were plotted for both V1 and LM; V1 texture response: 13.5% ± 0.28%, s.e.; LM texture response: 16.8% ± 0.31%, s.e.; $p < 10^{-6}$ all families, two-sided paired t-test Holm-Bonferroni corrected. Box plots indicate the median with a horizontal bar; the box height denotes the inter-quartile range (IQR, 1st and 3rd quartile) and the whiskers extend by 1.5 x IQR. Source data are provided as a Source Data file.

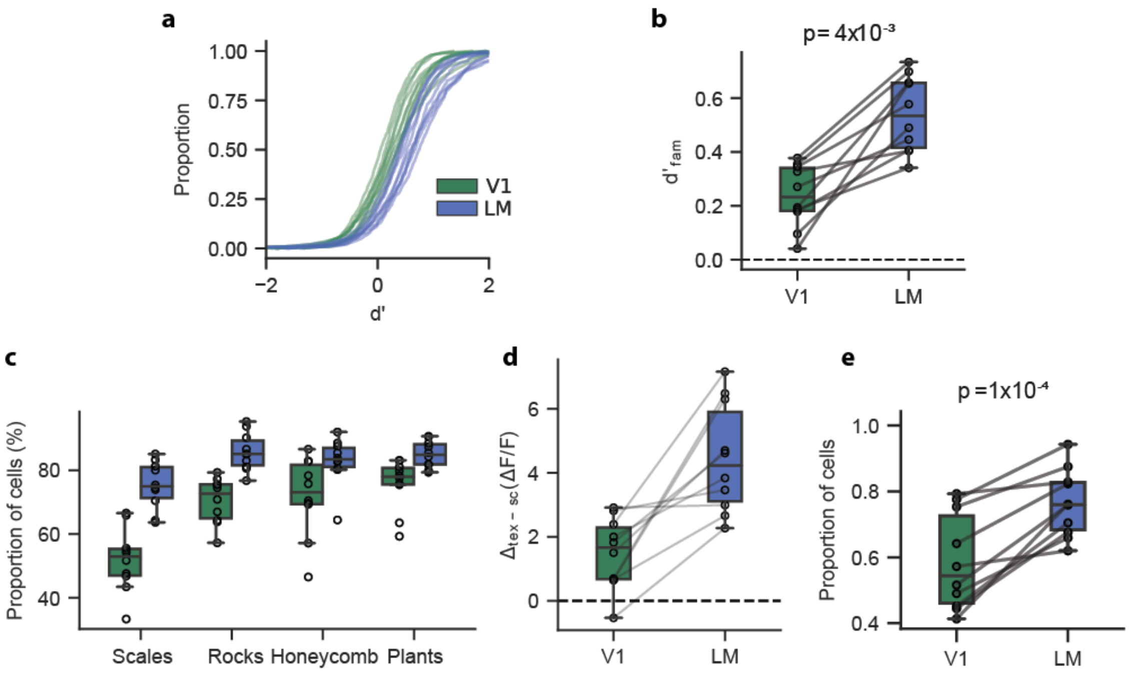

Supplementary Figure 6 | Single-cell responses to textures and scrambles.

a, Cumulative distributions of the neural discriminability values (d’) of cells (combining across experiments and averaging across texture families) in the discrimination of textures vs scrambles images; green for V1 and blue for LM. Each line is for one animal. **b**, Average d’ values for the discriminability between texture and scrambles combined across all families (each animal is a black dot; mean across cells and texture–scramble pairs) for V1 and LM; the p-value from a two-sided paired t-test across mice (n = 10); box plots indicate the median with a horizontal bar; the box height denotes the inter-quartile range (IQR, 1st and 3rd quartile) and the whiskers extend by 1.5 x IQR. **c**, The proportion of cells with a d’ > 0 is higher in LM than in V1 for each mouse (black dots) across all the texture–scramble pairs. P-values for each family: scales = 7.4×10^{-5}, rocks = 8.8×10^{-4}, honeycomb = 0.024, plants = 0.006, paired t-test. Proportions across families: V1: 67.5% ± 1.8% s.e., LM: 82.2% ± 1.4% s.e. Box plots as in (b). **d**, Average modulation difference for each animal (black dots, n = 10 mice, mean across all cells, exemplars, and families) for V1 and LM; V1 texture – scramble difference: 1.5% ± 0.25, LM: 3.5% ± 0.39, p = 0.002, two-sided paired t-test, n = 10. Box plots as in (b). **e**, Proportion of cells for each mouse (black dots) for which the regressive model based on the PS statistics had an explained variance EV ≥ 1%, separately for V1 and LM. The connecting lines are for the same animal; p-value, two-sided paired t-test; box plots as in (b). Source data are provided as a Source Data file.

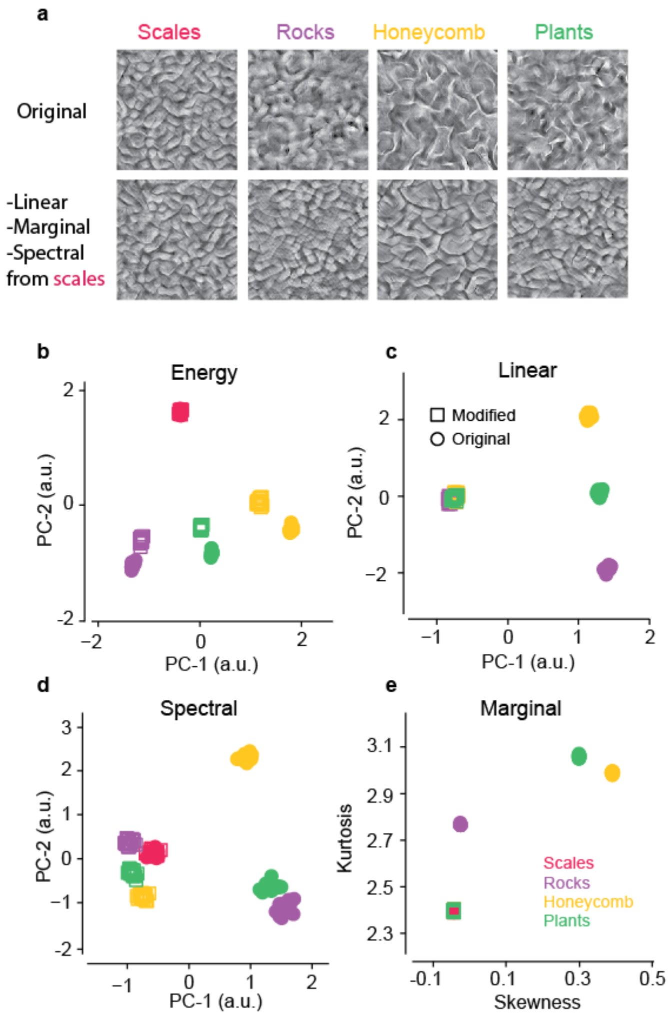

Supplementary Figure 7 | Synthesis of textures with equal linear, marginal, and spectral PS statistics.

a, Top row: textures synthesized using the Portilla-Simoncelli algorithm for four texture families. Bottom row: Textures synthesized using the same algorithm as in the top row, but the linear, marginal, and spectral statistics were constrained to be those of the scales family. Instead, the energy statistics were the original statistics of each of the four texture families. **b-e**, Two-dimensional PCA embedding of each of the four groups of image statistics (titles) for both the images synthesized with the original PS statistics (open dots) and the images whose linear, marginal and spectral statistics come from the scales family (open squares). Color code for texture families in the legend. Source data are provided as a Source Data file.

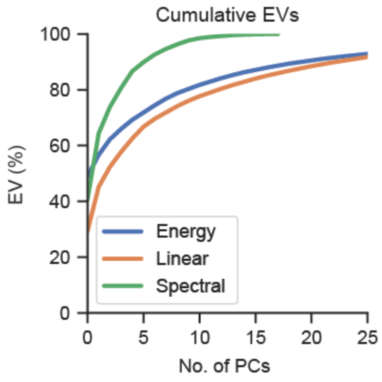

Supplementary Figure 8 | PCA decomposition of PS statistics.

PCA was used to reduce redundancies in the PS decomposition of the images. This panel shows the cumulative explained variance of the reduced PS statistics for the spectral, linear cross-correlation, and energy cross-correlation statistics. Source data are provided as a Source Data file.

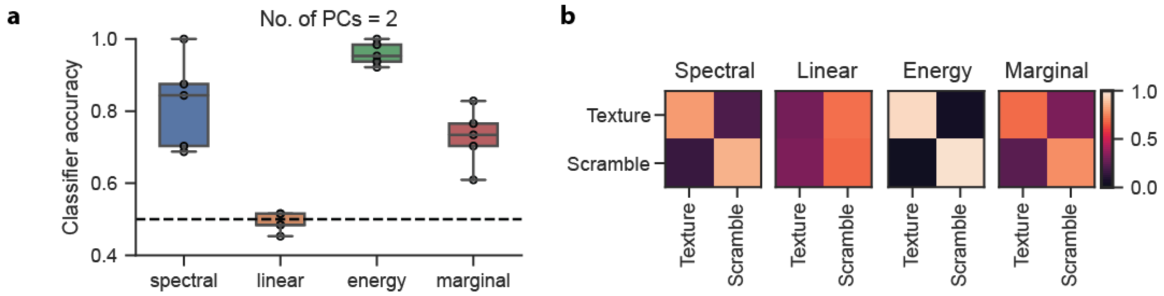

Supplementary Figure 9 | Texture vs scramble classifiers across a subset of PS image statistics.

a, Cross-validated performance of a binary linear classifier trained to discriminate between texture and scramble images (across all families and exemplars) based on different PS statistical groups (x-labels). The horizontal dotted line indicates chance-level accuracy. The energy cross-correlation statistics is the group of image statistics with the highest discriminability accuracy in a 2D-PCA embedding space; n = 5 classifiers from a 5-fold cross validated procedure. Box plots indicate the median with a horizontal bar; the box height denotes the inter-quartile range (IQR, 1st and 3rd quartile) and the whiskers extend by 1.5 x IQR. **b**, Confusion matrix for the texture-scramble classifiers is shown in (a) for each PS statistical group (titles). Source data are provided as a Source Data file.

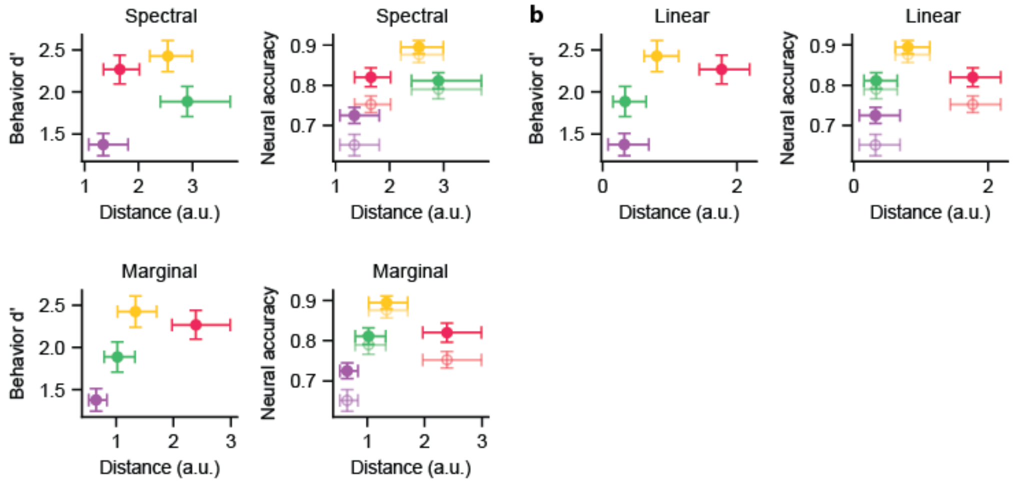

Supplementary Figure 10 | Neural, behavioral, and statistical distance measures for spectral, linear and marginal PS statistics.

a-c, Each panel illustrates the same concept as in Figure 4, that is, the relationship between neural accuracy, behavioral performance, and image statistics, but for the other three groups of PS image statistics: spectral, linear cross-correlation, and marginal statistics. The error bars for behavioral performance and classification accuracy are the standard error of the mean; the error bars for inter-cluster distances are the 99.7% confidence intervals with Šidák correction for multiple comparisons. Data points: behavior axis, n = 16 animals; neural accuracy axis, n = 10 mice (10 classifiers); distance axis, n = 1000 (bootstrap samples). Filled dots indicate: behavior axis, mean performance across all 16 mice; accuracy axis: mean classifier accuracy across all 10 mice; distance axis: mean of the bootstrapped distribution (1000 samples). Source data are provided as a Source Data file.

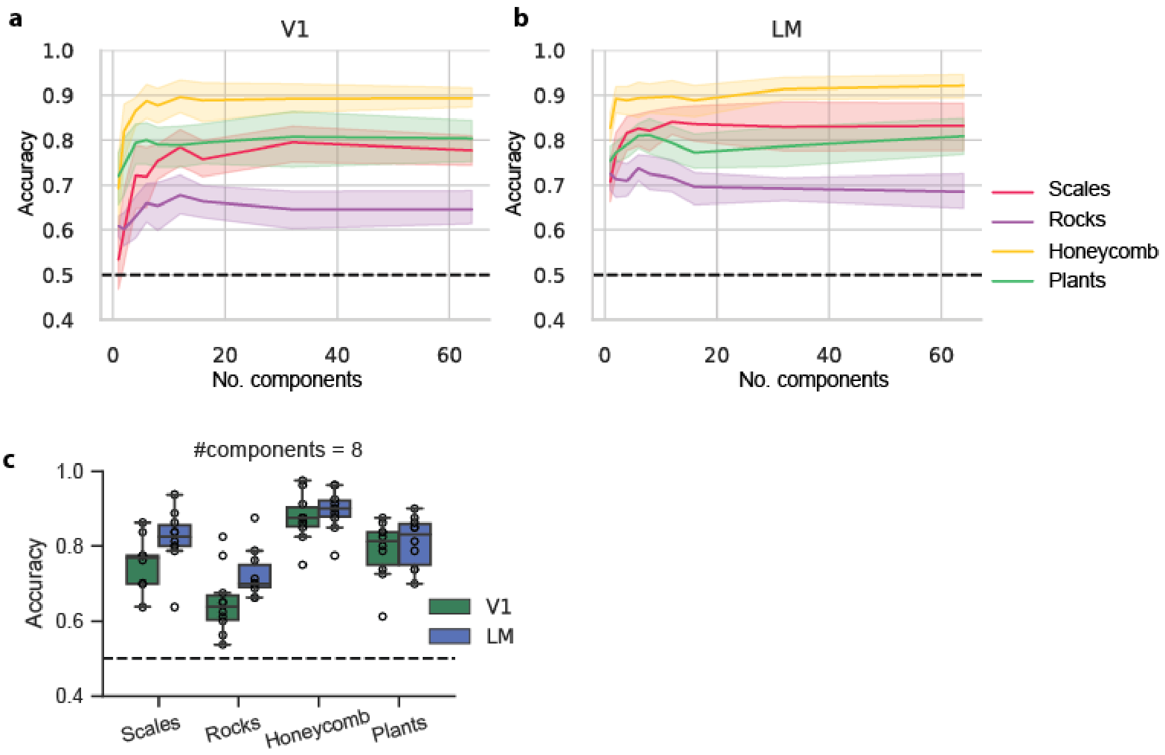

Supplementary Figure 11 | Binary classifiers of neural data for texture–scramble discrimination in V1 and LM.

a, The accuracy (fractional values) of a binary classifier trained on different pairs of texture–scramble families (legend in panel (b)) as a function of the number of components in the neural PCA space for V1. The shaded regions correspond to the 95% confidence interval for the average classification accuracy of all mice (n = 10). **b**, Same as in (a) but for LM. Fewer principal components (PCs) are needed in LM than in V1 to attain maximum performance: V1 accuracy, 12 dimensions: 78.6% ± 1.6% s.e.; LM accuracy, four dimensions: 78.6% ± 1.5%; $p < 0.05$ for mean accuracy of V1, 1-12 dimensions, vs LM, four dimensions, one-sided paired t-test, n = 10. **c**, The accuracy of the same binary classifier in (a, b) when using eight PCA components. One-sided one sample t-test relative to 50% accuracy: V1, scales $p = 1 \times 10^{-6}$; rocks $p = 5 \times 10^{-4}$; honeycomb $p = 2 \times 10^{-8}$; plants $p = 1 \times 10^{-6}$. LM: scales $p = 4 \times 10^{-7}$; rocks $p = 2 \times 10^{-6}$; honeycomb $p = 3 \times 10^{-9}$; plants $p = 2 \times 10^{-7}$. Source data are provided as a Source Data file.

Supplementary Table 1

Summary of the number of trials in a texture/scramble behavioral task

Mouse ID	Scales	Rocks	Honeycomb	Plants
20070	2189	n/a	1021	2029
20099	2015	1715	1122	2003
20100	2024	1979	1032	1677
20109	1853	1581	1000	1651
20117	1925	1938	1029	2213
21030	1471	1396	748	1682
21031	1816	1787	1056	1936
21032	n/a	n/a	1044	n/a
21033	1857	1560	1018	1856
21047	1839	1651	1985	756
21048	1002	1616	1852	1979
21049	n/a	n/a	871	n/a
21051	2059	1833	2049	870
21055	1666	1945	2059	864
21056	3712	1937	981	2040
21060	3845	2195	1118	2208
21061	1078	1892	2075	2172
21062	2042	2055	1962	1101
21064	3265	2332	2344	2260

Supplementary Table 2

Summary of the number of trials in a texture/texture behavioral task

Mouse ID	Honeycomb rocks	Honeycomb plants	Scales plants	Rocks plants	Honeycomb scales	Rocks scales
20100	2097	2046	1587	n/a	n/a	n/a
20117	n/a	1637	n/a	1618	1583	n/a
21030	n/a	n/a	1971	2060	2038	2087
21031	n/a	1615	1918	2162	2144	1956
21033	1916	n/a	1775	n/a	n/a	2206
21047	n/a	n/a	n/a	n/a	n/a	1992
21048	1669	n/a	n/a	n/a	1786	n/a
21049	1890	n/a	2008	3727	n/a	n/a
21051	n/a	n/a	n/a	n/a	2091	n/a
21055	1676	n/a	n/a	n/a	1705	n/a
21056	1960	2022	n/a	n/a	n/a	n/a
21060	1802	1940	n/a	n/a	n/a	n/a
21062	n/a	n/a	n/a	n/a	n/a	1963
21064	n/a	n/a	n/a	n/a	n/a	2123
21067	1797	n/a	n/a	1619	1813	n/a
21074	2138	n/a	n/a	n/a	n/a	n/a
